# Tetrel-Bond Interactions Involving Metallylenes TH_2_ (T = Si, Ge, Sn, Pb): Dual Binding Behavior

**DOI:** 10.3390/molecules28062577

**Published:** 2023-03-12

**Authors:** Yishan Chen, Lifeng Yao, Fan Wang

**Affiliations:** School of Chemistry & Environmental Science, Qujing Normal University, Qujing 655011, China

**Keywords:** intermolecular interaction, π-hole, σ-hole, MEP surfaces, NBO analysis

## Abstract

The dual binding behavior of the metallylenes TH_2_ (T = Si, Ge, Sn, Pb) with some selected Lewis acids (T’H_3_F, T’ = Si, Ge, Sn, Pb) and bases (N_2_, HCN, CO, and C_6_H_6_) has been investigated by using the high-level quantum chemical method. Two types (type-A and type-B) of tetrel-bonded complexes can be formed for TH_2_ due to their ambiphilic character. TH_2_ act as Lewis bases in type-A complexes, and they act as Lewis acids in type-B ones. CO exhibits two binding modes in the type-B complexes, one of which is TH_2_···CO and the other is TH_2_···OC. The TH_2_···OC complexes possess a weaker binding strength than the other type-B complexes. The TH_2_···OC complexes are referred to as the type-B2 complexes, and the other type-B complexes are referred to as the type-B1 complexes. The type-A complexes exhibit a relatively weak binding strength with *E*_int_ (interaction energy) values ranging from –7.11 to –15.55 kJ/mol, and the type-B complexes have a broad range of *E*_int_ values ranging from −9.45 to −98.44 kJ/mol. The *E*_int_ values of the type-A and type-B1 complexes go in the order SiH_2_ > GeH_2_ > SnH_2_ > PbH_2_. The AIM (atoms in molecules) analysis suggests that the tetrel bonds in type-A complexes are purely closed-shell interactions, and those in most type-B1 complexes have a partially covalent character. The EDA (Energy decomposition analysis) results indicate that the contribution values of the three energy terms go in the order electrostatic > dispersion > induction for the type-A and type-B2 complexes, and this order is electrostatic > induction > dispersion for the type-B1 complexes.

## 1. Introduction

Intermolecular interactions are a key issue for supermolecular chemistry because of their central role in molecular recognition [1,2,3]. A hydrogen bond is the most important intermolecular interaction and has been extensively applied in supermolecular systems [4]. A tetrel bond (TB) is another important intermolecular interaction, and the tetrel atoms, such as C and Si, serve as electron acceptors in TB interactions [5,6,7,8,9,10,11,12,13,14,15,16,17,18,19,20,21,22,23,24,25,26,27,28]. Its formation is ascribed to the areas of lower electronic density around tetrel atoms, and these areas are called σ-holes [29,30,31,32,33] or π-holes [34,35,36,37]. A σ-hole is an area of lower electronic density on the extension of a bond, and a π-hole is an area of a lower electronic density above and below a planar portion of a molecule. A number of investigations have been performed to understand the interplay between tetrel bonds and tetrel bonds or other types of noncovalent interactions [38,39,40,41,42,43,44,45,46,47,48]. The electron-rich species, such as lone pairs and π-systems, can be served as electron donors in TB interactions. The singlet carbenes can be expected to act as electron donors in TB interactions due to the existence of a lone-pair electron on the carbene C atom [49,50].

**Figure 1:**
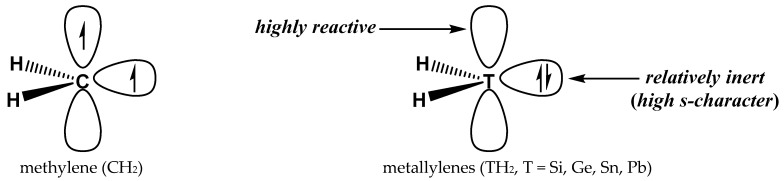
Ground-state structures of methylene and metallylenes.

The simplest carbene is methylene (CH_2_), and CH_2_ is too reactive to be isolated. The heavy-atom analogues of methylene, i.e., silylene (SiH_2_), germylene (GeH_2_), stannylene (SnH_2_), and plumbylene (PbH_2_), are the so-called metallylenes [51]. Figure 1 gives the ground-state structures of CH_2_ and TH_2_ (T = Si, Ge, Sn, Pb), and CH_2_ has a triplet ground state. Unlike CH_2_, the ground state is a singlet state for TH_2_. The singlet TH_2_ possesses two binding sites, namely, the lone pair electrons and the vacant p-orbital on the T atom. The lone pair can act as an electron donor (Lewis base), and the vacant p-orbital can serve as an electron acceptor (Lewis acid). Like CH_2_, TH_2_ is difficult to be isolated due to its especially high reactivity with other molecules [52,53,54,55,56,57,58,59,60,61,62,63,64]. This high reactivity is ascribed to the vacant p-orbital of TH_2_, and metallylenes serve as Lewis acids in these reactions. On the other hand, the lone pair of TH_2_ is generally expected to be relatively inert because the lone pair of TH_2_ exhibits higher s-character compared with CH_2_ [51]. A theoretical study of the possible dual binding behavior of metallylenes in TB interactions is necessary. First, the theoretical studies of TB interactions in which metallylenes act as Lewis bases are absent. Second, the theoretical studies of TB interactions in which metallylenes act as Lewis acids are sparse [65,66,67,68], and systematic studies involving all four metallylenes are still absent. Finally, it is informative to explore how the binding strength of TB interactions changes when the tetrel atoms become heavier. A comprehensive study of TB interactions involving metallylenes should be interesting and can be expected to provide some new insights into TB interactions.

In this study, we investigate the possible dual-binding behavior of metallylenes in TB interactions. We select T’H_3_F (T’ = Si, Ge, Sn, Pb) as electron acceptors to form the TB complexes with TH_2_ (T = Si, Ge, Sn, Pb). On the other hand, we select N_2_, HCN, CO, and C_6_H_6_ as electron donors to form the TB complexes with TH_2_. The molecular electrostatic potential (MEP) surface is useful for searching the approximate binding sites for intermolecular interactions. We first examine the MEP maps of the monomers to locate the possible binding sites for TB interactions, and then we discuss the geometries and binding strength of the TB complexes.

## 2. Results and Discussion

### 2.1. Geometries and MEP Surfaces of Monomers

**Figure 2:**
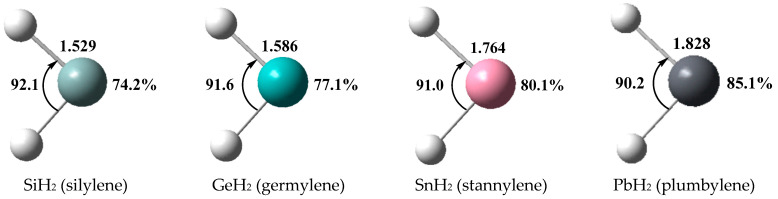
Optimized geometries of the metallylenes TH_2_ at the MP2/aug-cc-pVDZ level, distances in Å, angles in °, and the percentage of s-character of the lone pairs.

The optimized geometries of TH_2_ with the percentage of s-character of the corresponding lone pairs based on the Natural bond orbital (NBO) analysis are displayed in Figure 2. The values of H-T-H angles in metallylenes range from 90.2° to 92.1°, which are very close to 90° and are obviously smaller than that (101.6°) of H-C-H angle in singlet methylene. Additionally, the percentage of s-character of the lone pairs in metallylenes ranges from 74.2% to 85.1%, which is much larger than that in methylene (56.2%). These differences between metallylenes and methylene indicate that the heavier tetrel atoms, in contrast with the carbon atom, exhibit a weak ability to form hybrid orbitals and prefer to keep the ns^2^np^2^ valence electron configurations in metallylenes. It can also be observed that the s-character values increase with the increase of the T atomic number, suggesting that the lone pairs of metallylenes become more inert when the tetrel atoms become heavier. In other words, the electron-donating ability of TH_2_ should go in the order SiH_2_ > GeH_2_ > SnH_2_ > PbH_2_.

**Figure 3:**
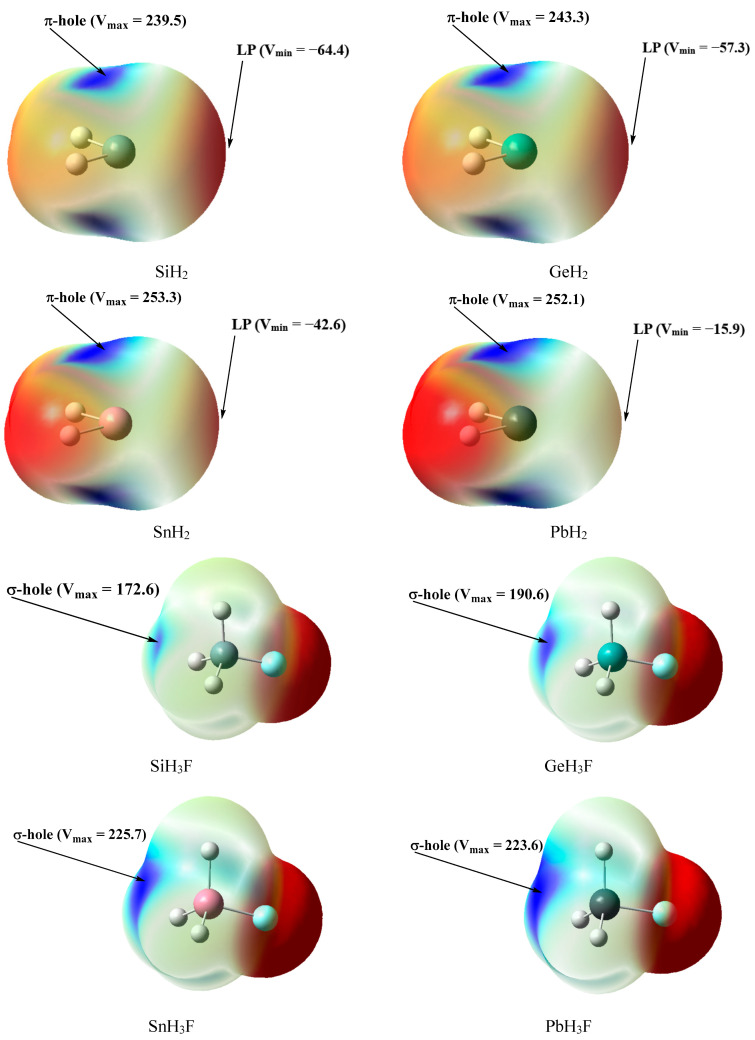
MEP surfaces of the monomers TH_2_ and T’H_3_F at the MP2/aug-cc-pVDZ level, *V*_S,max_, and *V*_S,min_ in kJ/mol.

The MEP surfaces of TH_2_ are illustrated in Figure 3, and the values of positive maxima (*V*_S,max_) and negative minima (*V*_S,min_) are also labeled. It can be observed that there exist two possible binding areas around T atoms, one of which is the π-hole area with a positive surface potential, and the other is the lone-pair (LP) area with a negative surface potential. The *V*_S,max_ values in different TH_2_ molecules are very close to each other, ranging from 239.5 to 253.3 kJ/mol. Unlike *V*_S,max_ values, the *V*_S,min_ values exhibit obvious differences, ranging from −15.9 to −64.4 kJ/mol. It should be noted that the absolute values of *V*_S,max_ are much larger than those of *V*_S,min_, which suggests that the electron-accepting ability of TH_2_ are much stronger than their electron-donating ability. The *V*_S,min_ values of TH_2_ go in the order SiH_2_ > GeH_2_ > SnH_2_ > PbH_2_, implying that the electron-donating ability of TH_2_ goes in the same order, which is consistent with the previous conclusion based on the s-character values of lone pairs. A similar phenomenon was observed for the N-heterocyclic carbene and its heavy-atom analogues [69].

**Figure 4:**
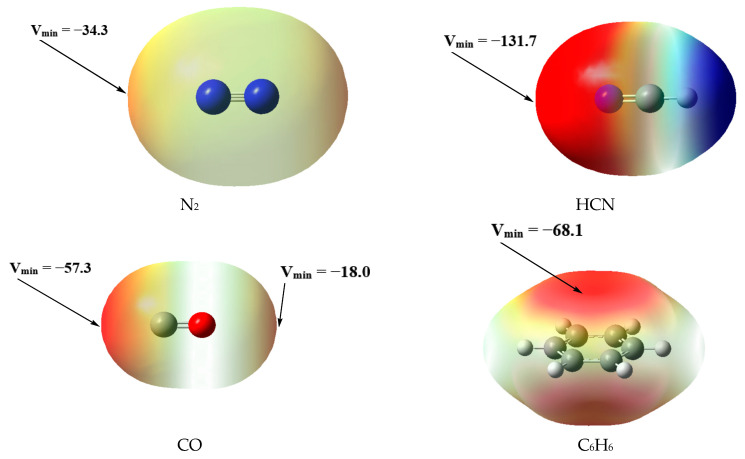
MEP surfaces of the selected Lewis bases at the MP2/aug-cc-pVDZ level, *V*_S,min_ in kJ/mol.

Two types of TB complexes can be formed for metallylenes due to their ambiphilic character, which we refer to as type-A and type-B for convenience. Metallylenes act as Lewis bases in type-A complexes, and they act as Lewis acids in type-B ones. T’H_3_F possess σ-holes and can act as Lewis acids in TB interactions, which can be expected to form the type-A TB complexes with TH_2_. Figure 3 gives the MEP surfaces of T’H_3_F, and their *V*_S,max_ values go in the order PbH_3_F ≈ SnH_3_F > GeH_3_F > SiH_3_F. We select N_2_, HCN, CO, and C_6_H_6_ as Lewis bases to form the type-B TB complexes with TH_2_, and their MEP surfaces are illustrated in Figure 4. N_2_, HCN, and CO possess the lone pairs, and C_6_H_6_ possesses the π-system, which can be served as electron donors in TB interactions. It can be observed that the *V*_S,min_ value (−131.7 kJ/mol) of HCN is much larger than that (−34.3 kJ/mol) of N_2_. Unlike N_2_, CO is a heteronuclear diatomic molecule and possesses two negative areas, one of which is around the C atom with a *V*_S,min_ value of −57.3 kJ/mol, and the other is around the O atom with a *V*_S,min_ value of −18.0 kJ/mol. The negative area of C_6_H_6_ is parallel to the benzene ring with a *V*_S,min_ value of −68.1 kJ/mol.

### 2.2. Type-A (σ-Hole Tetrel Bond) Complexes: TH_2_ Act as Lewis Bases

**Figure 5:**
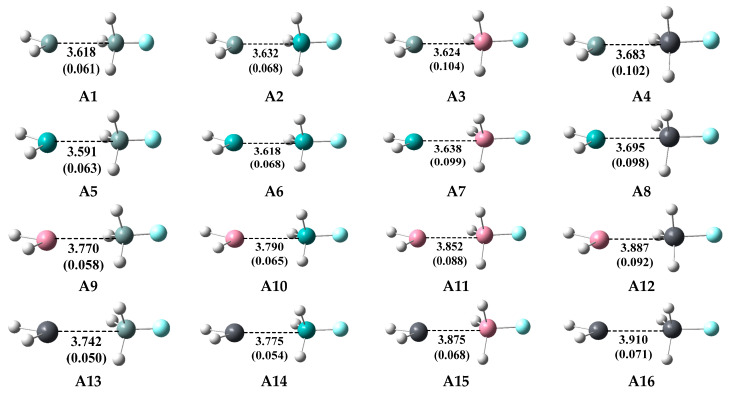
Optimized geometries of the type-A complexes at the MP2/aug-cc-pVDZ level, distances in Å, and WBI values (in parenthesis).

The type-A complexes are formed between TH_2_ and T’H_3_F, and the corresponding intermolecular interactions exist between two tetrel atoms, which are the σ-hole tetrel bonds. The geometries of the type-A complexes (**A1**–**A16**) optimized at the MP2/aug-cc-pVDZ level with the binding distances are displayed in Figure 5, and the corresponding Wiberg bond index (WBI) based on the NBO analysis are also labeled. Our previous studies indicate that the MP2/aug-cc-pVDZ level is more reasonable than MP2/aug-cc-pVTZ level for exploring the intermolecular interactions involving the heavy tetrel atoms. As a comparison, the geometries of these complexes were reoptimized at the MP2/aug-cc-pVTZ level, and the binding distances at the two levels are collected in Table 1. Additionally, the interaction energies (*E*_int_) of these complexes at the four different computational levels are also collected in Table 1. These four levels are referred to as L1 (MP2/aug-cc-pVDZ), L2 (CCSD (T)/aug-cc-pVTZ//MP2/aug-cc-pVDZ), L3 (MP2/aug-cc-pVTZ), and L4 (CCSD (T)/aug-cc-pVTZ//MP2/aug-cc-pVTZ), respectively.

It can be observed from Table 1 that the binding distances of all the sixteen type-A complexes at the L1 level are longer than those at the L3 level, which means that the complexes at the L3 level are overbound compared with the L1 level. It can also be found that the interaction energies at the L1 level are smaller than the corresponding values at the L2 level for all the type-A complexes, which indicates the L1 level underestimates the interaction energies compared with the L2 level. On the other hand, the L3 level overestimates the interaction energies compared with the L4 level. Furthermore, the interaction energies at the L2 level are larger than those at the L4 level in most cases, which suggests that the geometries optimized at the L1 level are more stable than those at the L3 level. The optimized geometries at the L1 level and the *E*_int_ values at the L2 level are employed in the following discussion.

Our preceding discussion indicates that the lone pairs of TH_2_ are relatively inert due to their high s-character, and the electron-donating ability of TH_2_ is expected to be weak. As expected, the type-A complexes indeed exhibit relatively weak binding strength with *E*_int_ values ranging from −7.11 to −15.55 kJ/mol. The type-A complexes possess relatively long T···T’ binding distances ranging from 3.618 to 3.910 Å and relatively small WBI values ranging from 0.050 to 0.104, suggesting that the tetrel bonds in TH_2_···T’H_3_F systems should be the noncovalent interactions, which is consistent with the Atoms in molecules (AIM) analysis as discussed below. The F-T’···T binding angles are linear for all the type-A complexes, ranging from 179° to 180°. For a given T’H_3_F, the *E*_int_ values of the type-A complexes go in the order SiH_2_ > GeH_2_ > SnH_2_ > PbH_2_. For example, the *E*_int_ values of the complexes **A4**, **A8**, **A12,** and **A16** are −15.42, −14.92, −13.25, and −9.86 kJ/mol, respectively. This order is in agreement with the LP *V*_S,min_ values of TH_2_, as shown in Figure 3. On the other hand, for a given TH_2_, the *E*_int_ values of the type-A complexes go in the order PbH_3_F ≈ SnH_3_F > GeH_3_F > SiH_3_F. For example, the *E*_int_ values of the complexes **A5**, **A6**, **A7,** and **A8** are −10.32, −11.08, −14.96, and −14.92 kJ/mol, respectively. This order is in agreement with the σ-hole *V*_S,max_ values of T’H_3_F, as shown in Figure 3.

**Figure 6:**
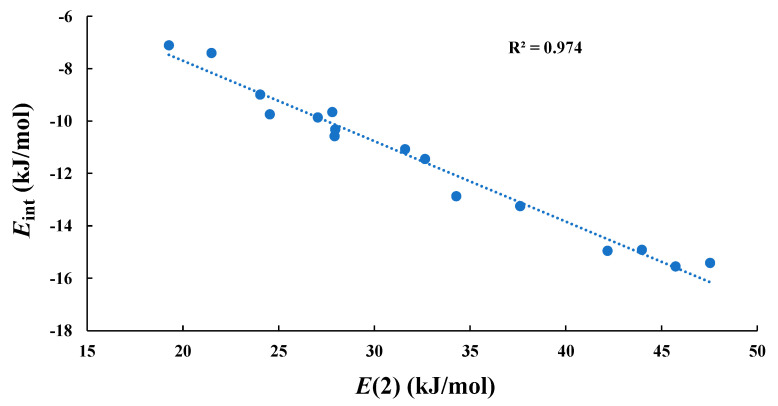
Correlation between the interaction energies (*E*_int_) and *E*(2) for the type-A complexes.

The NBO and AIM analysis results of the type-A complexes are listed in Table 2. NBO analysis shows that the dominant orbital interactions for the type-A complexes are LP (T)→σ* (T’-F), with the second-order perturbation stabilization energy *E*(2) values ranging from 19.27 to 47.53 kJ/mol. In fact, there exists a linear relationship between the *E*_int_ and *E*(2) values, with *R*^2^ = 0.974, as shown in Figure 6. In this study, the value of charge transfer (*q*_CT_) is the sum of the natural atomic charge over the TH_2_ molecule in the complexes. A positive *q*_CT_ represents that the direction of charge transfer is from TH_2_ to another molecule, and a negative *q*_CT_ represents the reverse direction. The *q*_CT_ values are positive for all the type-A complexes, indicating that TH_2_ act as Lewis bases in type-A complexes. AIM analysis indicates that there exist the intermolecular T···T’ bond critical points (BCP) in all the type-A complexes, and the electron density (*ρ*), Laplacian (∇^2^*ρ*), and energy density (*H*) at the BCP are listed in Table 2. The *ρ* values are smaller than 0.01 a.u. for all the type-A complexes. It can also be found that both ∇^2^*ρ* and *H* are positive for all the type-A complexes, suggesting that the tetrel bonds in type-A complexes are the purely closed-shell (noncovalent) interactions. The local kinetic energy density (*G*) and the local potential energy density (*V*) also might be used to analyze the electronic behavior at the intermolecular BCP. The values of *G* and *V* are listed in Table 2. The previous study indicates that the *G* and |*V*| values are increased with an increase of the stabilization energy (the absolute value of interaction energy) for the halogen-bonded complexes, implying that *G* and *V* might be considered as a measure of the strength of the intermolecular interaction [70]. Similar relations can also be found for most type-A complexes. For instance, there exist linear relationships between the |*E*_int_| and *G* or |*V*| values for the SiH_2_···T’H_3_F system, as shown in Figure S1.

**Figure 7:**
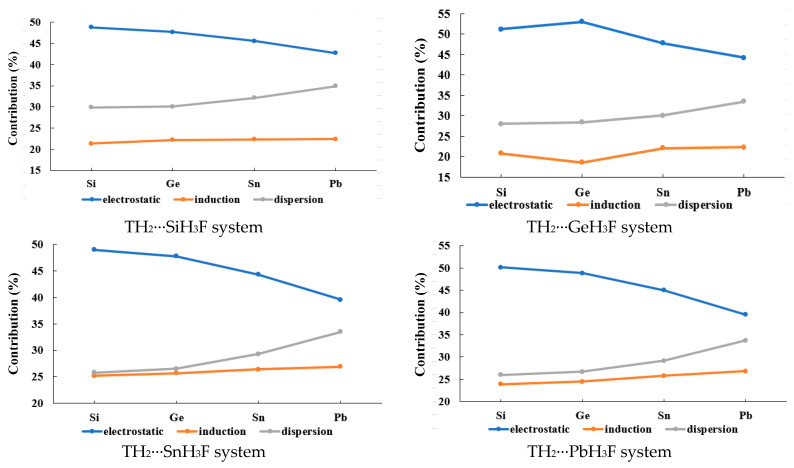
The changing trends of the contribution of the electrostatic, induction, and dispersion energy terms with the increase of the T atomic number for the type-A complexes.

The symmetry-adapted perturbation theory (SAPT) is a perturbation theory aimed specifically at calculating the interaction energy between two molecules. The result is obtained as a sum of separate corrections accounting for the electrostatic, induction, dispersion, and exchange contributions to interaction energy, so the SAPT decomposition facilitates the understanding and physical interpretation of results. Electrostatic energy arises from the Coulomb interaction between charge densities of isolated molecules. Induction energy is the energetic effect of mutual polarization between the two molecules. Dispersion energy is a consequence of intermolecular electron correlation, usually explained in terms of correlated fluctuations of electron density on both molecules. Exchange energy is a short-range repulsive effect that is a consequence of the Pauli exclusion principle. The Energy decomposition analysis (EDA) results of the type-A complexes are listed in Table 3, and the graphical changing trends of the contribution of the electrostatic, induction, and dispersion energy terms with the increase of the T atomic number are illustrated in Figure 7. The total interaction energy (*E*_tot_) values in Table 3 are similar to the *E*_int_ values at the L2 level in Table 1 for most complexes, suggesting that the EDA results are reasonable for the systems in this study. It can be observed that the contribution values of the three energy terms go in the order of electrostatic > dispersion > induction for all the type-A complexes. The contribution of the electrostatic term exhibits a decreasing trend, and that of the dispersion term exhibits an increasing trend with the increase of the T atomic number. Additionally, the contribution of the induction term is basically unchanged with the increase of the T atomic number.

### 2.3. Type-B (π-Hole Tetrel Bond) Complexes: TH_2_ Act as Lewis Acids

The metallylenes are the highly reactive Lewis acids and can interact with various Lewis bases. In this section, we select N_2_, HCN, CO, and C_6_H_6_ as Lewis bases to interact with TH_2_ to form the type-B complexes, which are the π-hole TB complexes. The binding distances and interaction energies of all the twenty type-B complexes (**B1**–**B20**) at different levels are collected in Table 4. Like type-A complexes, the binding distances of the type-B complexes at the L1 level are longer than those at the L3 level. It can also be found that the interaction energies of the type-B complexes at the L1 level are similar to those at the L2 level in most cases, but there exist relatively large differences between the L3 and L4 levels. The optimized geometries at the L1 level and the *E*_int_ values at the L2 level are employed in the following discussion.

**Figure 8:**
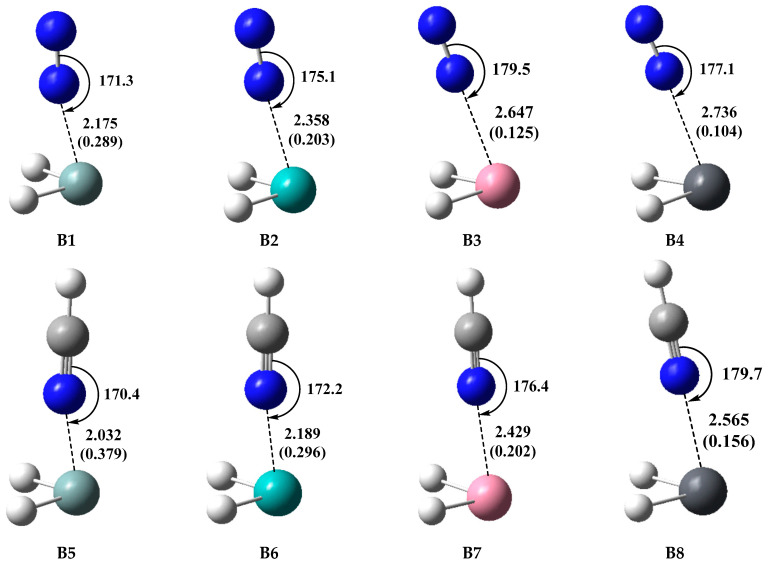
Optimized geometries of the type-B complexes involving N_2_ and HCN at the MP2/aug-cc-pVDZ level, distances in Å, angles in °, and WBI values (in parenthesis).

The optimized geometries of the type-B complexes (**B1**–**B8**) involving TH_2_ with N_2_ and HCN are shown in Figure 8. The formation of the complex SiH_2_···N_2_ (**B1**) was confirmed by the experimental study [61]. It should be noted that N_2_ is a rather weak Lewis base, and therefore the formation of **B1** reflects the high reactivity of SiH_2_ as a Lewis acid. The T···N binding distances range from 2.175 to 2.736 Å for the TH_2_···N_2_ complexes (**B1**–**B4**), with the N-N···T binding angles ranging from 171.3° to 179.5°. The complexes **B1**–**B4** possess larger *E*_int_ values ranging from −14.59 to −26.08 kJ/mol, with larger WBI values ranging from 0.104 to 0.289, compared with the type-A complexes. Like N_2_, HCN also uses the lone pair of the N atom as an electron-donor, but HCN is a stronger Lewis base compared with N_2_. HCN can form the TB complexes with various molecules. The C-N···T binding angles of the TH_2_···HCN complexes (**B5**–**B8**) range from 170.4° to 179.7°, which are similar to those of **B1**–**B4**, but **B5**–**B8** possess shorter T···N binding distances ranging from 2.032 to 2.565 Å compared with **B1**–**B4**. The *E*_int_ values of **B5**–**B8** range from −40.13 to −71.77 kJ/mol, which are nearly three times as large as those of **B1**–**B4**, and this difference in *E*_int_ values is in agreement with the *V*_S,min_ values of N_2_ and HCN, as shown in Figure 4. It can also be found that the WBI values of **B5**–**B8** are larger than the corresponding values of **B1**–**B4**. The *E*_int_ values of the complexes go in the order SiH_2_ > GeH_2_ > SnH_2_ > PbH_2_ for both the TH_2_···N_2_ and TH_2_···HCN systems.

**Figure 9:**
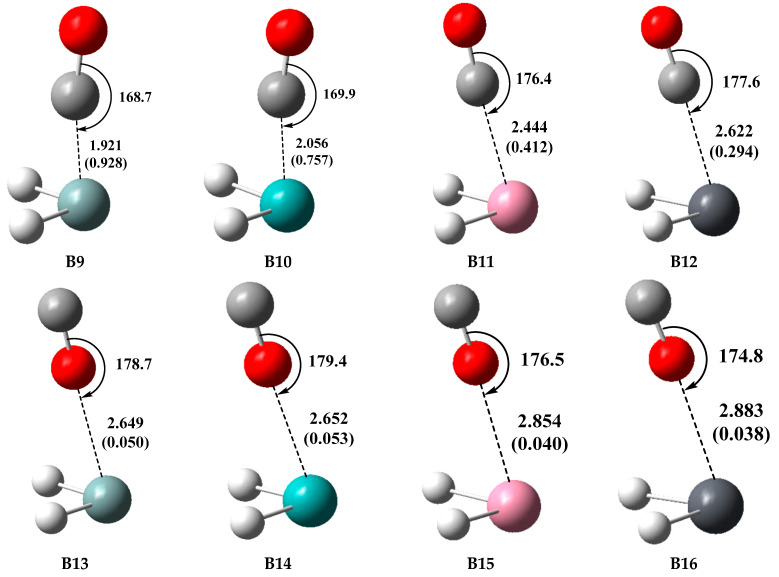
Optimized geometries of the type-B complexes involving CO at the MP2/aug-cc-pVDZ level, distances in Å, angles in °, and WBI values (in parenthesis).

The optimized geometries of the type-B complexes (**B9**–**B16**) involving TH_2_ with CO are shown in Figure 9, and the formation of the complex between SiH_2_ and CO was confirmed by the experimental study [61]. Unlike N_2_, CO is a heteronuclear diatomic molecule and exhibits two binding modes in the type-B complexes, one of which is TH_2_···CO and the other is TH_2_···OC. The fact that CO exhibits two binding modes and that complexes bound on the oxygen side are weaker has been previously reported [71]. The O-C···T binding angles of the TH_2_···CO complexes (**B9**–**B12**) range from 168.7° to 177.6°, which are similar to the C-O···T binding angles (174.8° to 179.4°) of the TH_2_···OC complexes (**B13**–**B16**), but **B9**–**B12** possess obviously shorter T···C binding distances ranging from 1.921 to 2.622 Å compared with the T···O binding distances (2.649 to 2.883 Å) of **B13**–**B16**. The WBI values of **B9**–**B12** range from 0.294 to 0.928, which are also much larger than those (0.038 to 0.053) of **B13**–**B16**. As expected, the TH_2_···CO complexes exhibit a stronger binding strength than the TH_2_···OC complexes, which is in agreement with the *V*_S,min_ values around C and O atoms of CO, as shown in Figure 4. The *E*_int_ value (−98.44 kJ/mol) of the complex SiH_2_···CO (**9**) is ten times as large as that (−9.45 kJ/mol) of the complex SiH_2_···OC (**13**), and **9** also possesses a rather large WBI value of 0.928, suggesting that **9** has a partially covalent character, which is in agreement with the AIM analysis as discussed below. Considering that the *V*_S,min_ value (−57.3 kJ/mol) around the C atom of CO is not large, it is somewhat surprising that **9** possesses such a high *E*_int_ value. The TH_2_···CO complexes have a broad range of *E*_int_ values ranging from −32.10 to −98.44 kJ/mol, and in contrast, the TH_2_···OC complexes have a very narrow range of *E*_int_ values ranging from −9.45 to −10.07 kJ/mol. The TH_2_···OC complexes possess a weaker binding strength than the other type-B complexes and exhibit a different binding behavior, as discussed below. We refer to the TH_2_···OC complexes as the type-B2 complexes and refer to the other type-B complexes as the type-B1 complexes in the following discussions. Like the TH_2_···N_2_ and TH_2_···HCN systems, the *E*_int_ values of the TH_2_···CO system go in the order SiH_2_ > GeH_2_ > SnH_2_ > PbH_2_.

**Figure 10:**
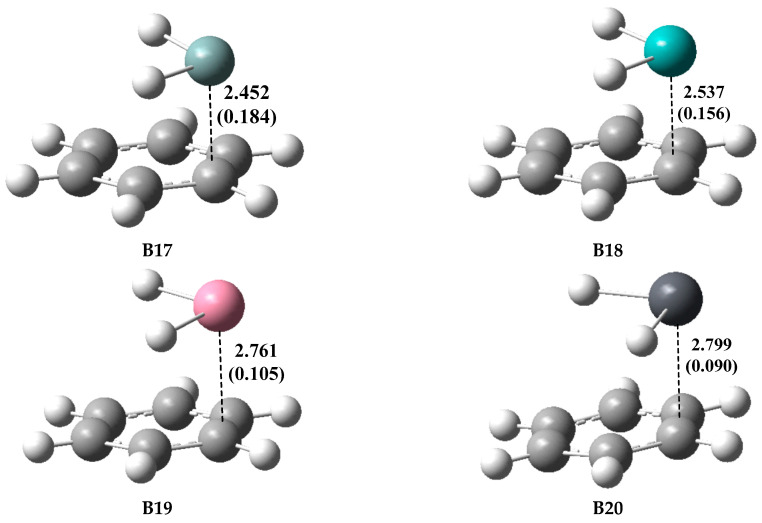
Optimized geometries of the type-B complexes involving C_6_H_6_ at the MP2/aug-cc-pVDZ level, distances in Å, angles in °, and WBI values (in parenthesis).

The optimized geometries of the type-B complexes (**B17**–**B20**) involving TH_2_ with C_6_H_6_ are shown in Figure 10. Unlike the other three Lewis bases for which the lone pairs are used as the electron-donors, C_6_H_6_ uses the π-system as an electron-donor to form the type-B complexes with TH_2_. As expected, TH_2_ molecules are parallel to the benzene ring in these π-hole TB complexes. **B17**–**B20** possess the T···C binding distances ranging from 2.452 to 2.799 Å, with the WBI values ranging from 0.090 to 0.184. The *E*_int_ values of **B17**–**B20** range from −32.35 to −43.64 kJ/mol, which are larger than those of the TH_2_···N_2_ system but smaller than those of the TH_2_···HCN system. Like the other type-B1 complexes, the *E*_int_ values of the complexes go in the order SiH_2_ > GeH_2_ > SnH_2_ > PbH_2_ for the TH_2_···C_6_H_6_ system.

As mentioned before, the relative binding strength of the type-A complexes can be clarified by the MEP maps of the corresponding monomers in a reasonable way, but this explanation is not applicable to the type-B1 complexes. Considering that TH_2_ have a narrow range of *V*_S,max_ values ranging from 239.5 to 253.3 kJ/mol, one may expect that for a given Lewis base, the *E*_int_ values of the type-B complexes should be very close to each other. However, for a given Lewis base, the type-B1 complexes have a relatively broad range of *E*_int_ values, and the *E*_int_ values go in the order SiH_2_ > GeH_2_ > SnH_2_ > PbH_2_. Additionally, the *E*_int_ values of the type-B1 complexes go in the order CO > HCN > C_6_H_6_ > N_2_ for SiH_2_ and GeH_2_; HCN > CO > C_6_H_6_ > N_2_ for SnH_2_; and HCN > C_6_H_6_ ≈ CO > N_2_ for PbH_2_. Unlike the type-B1 complexes, the *E*_int_ values of the type-B2 complexes are very close to each other, which is consistent with the narrow range of *V*_S,max_ values of TH_2_.

The NBO and AIM analysis results of the type-B complexes are listed in Table 5. NBO analysis shows that the dominant orbital interactions for the type-B complexes are LP (B)→LP * (T) (B = N, C, and O) and π (C = C)→LP * (T) with a very broad range of *E*(2) values ranging from 49.45 to 1488.16 kJ/mol. The *E*(2) values of the type-B1 complexes are larger than those of the type-B2 and type-A complexes. The *q*_CT_ values are negative for all the type-B complexes, indicating that TH_2_ act as Lewis acids in type-B complexes. AIM analysis indicates that there exist the intermolecular T···B (B = N, C, and O) bond critical points in the type-B complexes. Like *E*(2) values, the *ρ* values of the type-B1 complexes are larger than those of the type-B2 and type-A complexes. It can also be found that ∇^2^*ρ* are positive and *H* are negative for most type-B1 complexes, suggesting that these complexes have a partially covalent character. There exists a linear relationship between the |*E*_int_| and *G* values and an approximately linear relationship between the |*E*_int_| and |*V*| values for the TH_2_···CO system, as shown in Figure S2.

**Figure 11:**
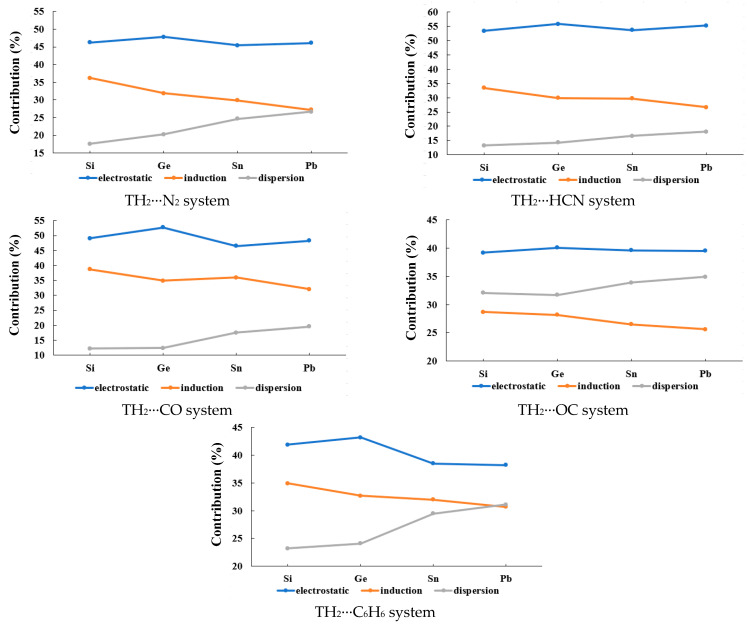
The changing trends of the contribution of the electrostatic, induction, and dispersion energy terms with the increase of the T atomic number for the type-B complexes.

The EDA results of the type-B complexes are listed in Table 6, and the graphical illustration is shown in Figure 11. The contribution of the electrostatic term exhibits a fluctuating trend (first increase and then decrease) with the increase of the T atomic number. On the other hand, the contribution of the induction term exhibits a decreasing trend, and that of the dispersion term exhibits an increasing trend with the increase of the T atomic number. It can also be observed that the contribution values of the three energy terms go in the order electrostatic > induction > dispersion for the type-B1 complexes, and this order is electrostatic > dispersion > induction for the type-B2 complexes.

## 3. Computational Methods

The geometries of all the monomers and complexes investigated in this study were fully optimized at the MP2 level of theory using the Gaussian 09 programs [72]. The aug-cc-pVDZ-PP basis set, which uses pseudopotentials to describe the inner core orbitals [73], was applied to Sn and Pb atoms, whereas aug-cc-pVDZ was used for else atoms. The vibrational frequencies were calculated for all the optimized geometries at the same level. As a comparison, the geometries of all the complexes were reoptimized at the MP2/aug-cc-pVTZ (aug-cc-pVTZ-PP for Sn and Pb atoms) level. Single-point energy calculations were performed at the CCSD (T)/aug-cc-pVTZ level to obtain more accurate energies. Interaction energy is defined as the difference between the energy of the complex and the sum of the monomers retaining their internal geometries as in the complex. Basis set superposition error (BSSE) correction was carried out following the counterpoise (CP) method [74]. AIM analysis [75] and MEP calculation were carried out using the Multiwfn program [76], and the MEP maps were generated on a 0.001 a.u. isodensity surface and plotted using GaussView software [77]. NBO analysis [78] was performed via the procedures contained within Gaussian 09. Energy decomposition analysis (EDA) based on symmetry-adapted perturbation theory (SAPT) [79] was performed at the sapt2+dmp2/aug-cc-pVDZ level using the Psi4 package [80].

## 4. Conclusions

In this study, the dual binding behavior of the metallylenes TH_2_ with some selected Lewis acids and bases has been investigated. Two types (type-A and type-B) of TB complexes can be formed for TH_2_ due to their ambiphilic character. TH_2_ act as Lewis bases in type-A complexes, and they act as Lewis acids in type-B ones. T’H_3_F possess σ-holes and can act as Lewis acids to form the type-A complexes with TH_2_, which are the σ-hole TB complexes. N_2_, HCN, CO, and C_6_H_6_ possess the lone pair or π-system and can act as Lewis bases to form the type-B complexes with TH_2_, which are the π-hole TB complexes. CO exhibits two binding modes in the type-B complexes, one of which is TH_2_···CO and the other is TH_2_···OC. The TH_2_···OC complexes possess a weaker binding strength than the other type-B complexes. The TH_2_···OC complexes are referred to as the type-B2 complexes, and the other type-B complexes are referred to as the type-B1 complexes. The type-A complexes exhibit a relatively weak binding strength with *E*_int_ values ranging from −7.11 to −15.55 kJ/mol. The type-B complexes have a broad range of *E*_int_ values ranging from −9.45 to −98.44 kJ/mol, and the *E*_int_ values of the type-B1 complexes are larger than those of the type-B2 and type-A complexes. For a given T’H_3_F, the *E*_int_ values of the type-A complexes go in the order SiH_2_ > GeH_2_ > SnH_2_ > PbH_2_, and for a given TH_2_, the *E*_int_ values of the type-A complexes go in the order PbH_3_F ≈ SnH_3_F > GeH_3_F > SiH_3_F, which can be clarified by the MEP maps of TH_2_ and T’H_3_F in a reasonable way. For a given Lewis base, the type-B1 complexes have a relatively broad range of *E*_int_ values, and the *E*_int_ values go in the order SiH_2_ > GeH_2_ > SnH_2_ > PbH_2_. Additionally, the *E*_int_ values of the type-B1 complexes go in the order CO > HCN > C_6_H_6_ > N_2_ for SiH_2_ and GeH_2_; HCN > CO > C_6_H_6_ > N_2_ for SnH_2_; and HCN > C_6_H_6_ ≈ CO > N_2_ for PbH_2_. Unlike the type-B1 complexes, the *E*_int_ values of the type-B2 complexes are very close to each other, which is consistent with the narrow range of *V*_S,max_ values of TH_2_. The AIM analysis suggests that the tetrel bonds in type-A complexes are the purely closed-shell interactions, and those in most type-B1 complexes have a partially covalent character. The EDA results indicate that the contribution values of the three energy terms go in the order of electrostatic > dispersion > induction for the type-A and type-B2 complexes, and this order is electrostatic > induction > dispersion for the type-B1 complexes.

## Figures and Tables

**Table 1:** Binding distance at MP2/aug-cc-pVDZ and MP2/aug-cc-pVTZ (in parentheses) levels (*R*, in Å) and interaction energy at various levels (*E*_int_, in kJ/mol) for type-A complexes.

Complex	*R*	*E*_int_ ^a^			
		L1	L2	L3	L4
**A1** (SiH_2_···SiH_3_F)	3.618 (3.505)	−9.07	−10.58	−10.91	−9.95
**A2** (SiH_2_···GeH_3_F)	3.632 (3.516)	−10.37	−11.45	−12.00	−10.78
**A3** (SiH_2_···SnH_3_F)	3.624 (3.493)	−14.25	−15.55	−17.68	−15.68
**A4** (SiH_2_···PbH_3_F)	3.683 (3.575)	−14.46	−15.42	−17.68	−15.38
**A5** (GeH_2_···SiH_3_F)	3.591 (3.437)	−8.32	−10.32	−10.53	−9.41
**A6** (GeH_2_···GeH_3_F)	3.618 (3.465)	−9.53	−11.08	−11.45	−10.07
**A7** (GeH_2_···SnH_3_F)	3.638 (3.463)	−13.13	−14.96	−16.89	−14.92
**A8** (GeH_2_···PbH_3_F)	3.695 (3.545)	−13.38	−14.92	−17.01	−14.71
**A9** (SnH_2_···SiH_3_F)	3.770 (3.634)	−6.94	−8.99	−8.82	−7.90
**A10** (SnH_2_···GeH_3_F)	3.790 (3.660)	−7.98	−9.66	−9.66	−8.53
**A11** (SnH_2_···SnH_3_F)	3.852 (3.672)	−10.91	−12.87	−13.88	−12.29
**A12** (SnH_2_···PbH_3_F)	3.887 (3.709)	−11.45	−13.25	−14.67	−12.54
**A13** (PbH_2_···SiH_3_F)	3.742 (3.537)	−4.68	−7.11	−5.81	−4.93
**A14** (PbH_2_···GeH_3_F)	3.775 (3.556)	−5.27	−7.40	−5.64	−4.51
**A15** (PbH_2_···SnH_3_F)	3.875 (3.661)	−7.40	−9.74	−9.82	−8.53
**A16** (PbH_2_···PbH_3_F)	3.910 (3.700)	−7.69	−9.86	−10.49	−8.61

^a^ L1: MP2/aug-cc-pVDZ; L2: CCSD(T)/aug-cc-pVTZ//MP2/aug-cc-pVDZ; L3: MP2/aug-cc-pVTZ; L4: CCSD(T)/aug-cc-pVTZ//MP2/aug-cc-pVTZ.

**Table 2:** Second-order perturbation stabilization energy (*E*(2), in kJ/mol), charge transfer (qCT, in e), electron density (*ρ*, in a.u.), Laplacian (∇2*ρ*, in a.u.), energy density (H, in a.u.), local kinetic energy density (G, in a.u.) and potential energy density (V, in a.u.) at the BCP for type-A complexes.

Complex	Orbital Interaction	*E*(2)	*q*_CT_	*ρ*	∇ ^2^ *ρ*	*H*	*G*	*V*
**A1** (SiH_2_···SiH_3_F)	LP (Si)→σ* (Si-F)	27.92	0.0340	0.0077	0.0179	0.0005	0.0040	−0.0035
**A2** (SiH_2_···GeH_3_F)	LP (Si)→σ* (Ge-F)	32.65	0.0389	0.0080	0.0183	0.0005	0.0041	−0.0036
**A3** (SiH_2_···SnH_3_F)	LP (Si)→σ* (Sn-F)	45.73	0.0614	0.0098	0.0204	0.0004	0.0047	−0.0043
**A4** (SiH_2_···PbH_3_F)	LP (Si)→σ* (Pb-F)	47.53	0.0595	0.0099	0.0219	0.0006	0.0048	−0.0043
**A5** (GeH_2_···SiH_3_F)	LP (Ge)→σ* (Si-F)	27.96	0.0355	0.0082	0.0189	0.0005	0.0043	−0.0038
**A6** (GeH_2_···GeH_3_F)	LP (Ge)→σ* (Ge-F)	31.60	0.0393	0.0083	0.0190	0.0005	0.0042	−0.0037
**A7** (GeH_2_···SnH_3_F)	LP (Ge)→σ* (Sn-F)	42.18	0.0587	0.0097	0.0203	0.0004	0.0047	−0.0042
**A8** (GeH_2_···PbH_3_F)	LP (Ge)→σ* (Pb-F)	43.97	0.0571	0.0098	0.0219	0.0006	0.0049	−0.0043
**A9** (SnH_2_···SiH_3_F)	LP (Sn)→σ* (Si-F)	24.04	0.0339	0.0074	0.0162	0.0004	0.0036	−0.0032
**A10** (SnH_2_···GeH_3_F)	LP (Sn)→σ* (Ge-F)	27.80	0.0385	0.0076	0.0163	0.0004	0.0036	−0.0032
**A11** (SnH_2_···SnH_3_F)	LP (Sn)→σ* (Sn-F)	34.28	0.0540	0.0083	0.0162	0.0004	0.0037	−0.0033
**A12** (SnH_2_···PbH_3_F)	LP (Sn)→σ* (Pb-F)	37.62	0.0555	0.0086	0.0180	0.0005	0.0040	−0.0035
**A13** (PbH_2_···SiH_3_F)	LP (Pb)→σ* (Si-F)	19.27	0.0298	0.0074	0.0173	0.0005	0.0039	−0.0034
**A14** (PbH_2_···GeH_3_F)	LP (Pb)→σ* (Ge-F)	21.49	0.0327	0.0075	0.0171	0.0005	0.0038	−0.0033
**A15** (PbH_2_···SnH_3_F)	LP (Pb)→σ* (Sn-F)	24.54	0.0423	0.0077	0.0161	0.0004	0.0036	−0.0032
**A16** (PbH_2_···PbH_3_F)	LP (Pb)→σ* (Pb-F)	27.04	0.0437	0.0080	0.0179	0.0006	0.0039	−0.0034

**Table 3:** Decomposition of the total interaction energy (Etot) for type-A complexes into electrostatic (Eele), induction (Eind), dispersion (Edisp), and exchange (Eex) energy terms. All energies in kJ/mol. The relative values in percent represent the contribution of electrostatic, induction, and dispersion energy terms to the sum of all the three energy terms.

Complex	*E*_ele_	%*E*_ele_	*E*_ind_	%*E*_ind_	*E*_disp_	%*E*_disp_	*E*_ex_	*E*_tot_
**A1** (SiH_2_···SiH_3_F)	−20.52	48.8	−8.95	21.3	−12.58	29.9	31.94	−10.12
**A2** (SiH_2_···GeH_3_F)	−23.03	51.2	−9.36	20.8	−12.58	28.0	33.94	−11.04
**A3** (SiH_2_···SnH_3_F)	−26.75	49.0	−13.75	25.2	−14.04	25.8	38.87	−15.68
**A4** (SiH_2_···PbH_3_F)	−25.41	50.1	−12.12	23.9	−13.21	26.0	35.11	−15.63
**A5** (GeH_2_···SiH_3_F)	−21.23	47.7	−9.86	22.2	−13.38	30.1	34.82	−9.66
**A6** (GeH_2_···GeH_3_F)	−24.49	53.0	−8.61	18.6	−13.13	28.4	35.82	−10.41
**A7** (GeH_2_···SnH_3_F)	−25.33	47.8	−13.63	25.7	−14.04	26.5	38.29	−14.71
**A8** (GeH_2_···PbH_3_F)	−24.12	48.8	−12.12	24.5	−13.21	26.7	34.74	−14.71
**A9** (SnH_2_···SiH_3_F)	−19.06	45.6	−9.32	22.3	−13.42	32.1	33.61	−8.19
**A10** (SnH_2_···GeH_3_F)	−21.07	47.8	−9.74	22.1	−13.29	30.1	35.28	−8.82
**A11** (SnH_2_···SnH_3_F)	−20.36	44.3	−12.12	26.4	−13.46	29.3	33.61	−12.33
**A12** (SnH_2_···PbH_3_F)	−20.06	45.0	−11.50	25.8	−13.00	29.2	31.94	−12.62
**A13** (PbH_2_···SiH_3_F)	−16.22	42.7	−8.49	22.4	−13.25	34.9	32.02	−5.94
**A14** (PbH_2_···GeH_3_F)	−17.01	44.2	−8.61	22.3	−12.92	33.5	32.35	−6.19
**A15** (PbH_2_···SnH_3_F)	−14.67	39.6	−9.99	26.9	−12.41	33.5	28.38	−8.69
**A16** (PbH_2_···PbH_3_F)	−14.13	39.5	−9.57	26.8	−12.04	33.7	26.96	−8.78

**Table 4:** Binding distance at MP2/aug-cc-pVDZ and MP2/aug-cc-pVTZ (in parentheses) levels (*R*, in Å) and interaction energy at various levels (*E*_int_, in kJ/mol) for type-B complexes.

Complex	*R*	*E*_int_ ^a^			
		L1	L2	L3	L4
**B1** (SiH_2_···N_2_)	2.175 (2.042)	−24.95	−26.08	−35.70	−25.83
**B2** (GeH_2_···N_2_)	2.358 (2.211)	−19.98	−19.73	−25.16	−17.10
**B3** (SnH_2_···N_2_)	2.647 (2.549)	−16.34	−15.84	−20.06	−14.55
**B4** (PbH_2_···N_2_)	2.736 (2.643)	−14.50	−14.59	−17.77	−12.67
**B5** (SiH_2_···HCN)	2.032 (1.951)	−68.09	−71.77	−85.06	−73.36
**B6** (GeH_2_···HCN)	2.189 (2.087)	−55.22	−55.64	−65.42	−54.97
**B7** (SnH_2_···HCN)	2.429 (2.373)	−47.23	−46.77	−54.51	−46.61
**B8** (PbH_2_···HCN)	2.565 (2.485)	−40.46	−40.13	−45.85	−38.54
**B9** (SiH_2_···CO)	1.921 (1.889)	−93.30	−98.44	−113.11	−97.77
**B10** (GeH_2_···CO)	2.056 (1.989)	−64.83	−68.55	−81.26	−67.26
**B11** (SnH_2_···CO)	2.444 (2.347)	−38.00	−40.80	−48.53	−40.00
**B12** (PbH_2_···CO)	2.622 (2.498)	−29.80	−32.10	−37.29	−29.93
**B13** (SiH_2_···OC)	2.649 (2.581)	−8.03	−9.45	−9.95	−9.03
**B14** (GeH_2_···OC)	2.652 (2.593)	−7.86	−9.61	−9.66	−8.95
**B15** (SnH_2_···OC)	2.854 (2.769)	−7.65	−9.70	−9.24	−8.95
**B16** (PbH_2_···OC)	2.883 (2.785)	−7.65	−10.07	−9.28	−8.86
**B17** (SiH_2_···C_6_H_6_)	2.452 (2.381)	−47.15	−43.64	−57.06	−42.64
**B18** (GeH_2_···C_6_H_6_)	2.537 (2.446)	−42.76	−39.29	−51.21	−36.74
**B19** (SnH_2_···C_6_H_6_)	2.761 (2.700)	−37.75	−34.99	−44.73	−32.65
**B20** (PbH_2_···C_6_H_6_)	2.799 (2.746)	−34.49	−32.35	−41.72	−28.47

^a^ L1: MP2/aug-cc-pVDZ; L2: CCSD(T)/aug-cc-pVTZ//MP2/aug-cc-pVDZ; L3: MP2/aug-cc-pVTZ; L4: CCSD(T)/aug-cc-pVTZ//MP2/aug-cc-pVTZ.

**Table 5:** Second-order perturbation stabilization energy (*E*(2), in kJ/mol), charge transfer (*q*_CT_, in e), electron density (*ρ*, in a.u.), Laplacian (∇^2^*ρ*, in a.u.), energy density (*H*, in a.u.), local kinetic energy density (*G*, in a.u.) and potential energy density (*V*, in a.u.) at the BCP for type-B complexes.

Complex	Orbital Interaction	*E*(2)	*q*_CT_	*ρ*	∇ ^2^ *ρ*	*H*	*G*	*V*
**B1** (SiH_2_···N_2_)	LP (N)→LP* (Si)	362.57	−0.0592	0.0401	0.0605	−0.0124	0.0275	−0.0399
**B2** (GeH_2_···N_2_)	LP (N)→LP* (Ge)	238.01	−0.0538	0.0341	0.1253	−0.0021	0.0334	−0.0354
**B3** (SnH_2_···N_2_)	LP (N)→LP* (Sn)	135.10	−0.0384	0.0233	0.0880	0.0007	0.0213	−0.0205
**B4** (PbH_2_···N_2_)	LP (N)→LP* (Pb)	113.78	−0.0346	0.0226	0.0895	0.0017	0.0207	−0.0191
**B5** (SiH_2_···HCN)	LP (N)→LP* (Si)	520.33	−0.0898	0.0522	0.1647	−0.0144	0.0556	−0.0700
**B6** (GeH_2_···HCN)	LP (N)→LP* (Ge)	379.46	−0.0860	0.0514	0.1808	−0.0097	0.0549	−0.0645
**B7** (SnH_2_···HCN)	LP (N)→LP* (Sn)	229.69	−0.0639	0.0387	0.1529	−0.0014	0.0397	−0.0411
**B8** (PbH_2_···HCN)	LP (N)→LP* (Pb)	178.74	−0.0534	0.0343	0.1357	0.0007	0.0332	−0.0325
**B9** (SiH_2_···CO)	LP (C)→LP* (Si)	1488.16	−0.1009	0.0762	0.3377	−0.0215	0.1059	−0.1274
**B10** (GeH_2_···CO)	LP (C)→LP* (Ge)	1092.61	−0.1294	0.0790	0.2162	−0.0305	0.0846	−0.1151
**B11** (SnH_2_···CO)	LP (C)→LP* (Sn)	465.11	−0.1125	0.0424	0.1484	−0.0038	0.0409	−0.0446
**B12** (PbH_2_···CO)	LP (C)→LP* (Pb)	317.68	−0.0973	0.0341	0.1218	−0.0001	0.0306	−0.0307
**B13** (SiH_2_···OC)	LP (O)→LP* (Si)	63.16	−0.0187	0.0147	0.0385	−0.0012	0.0108	−0.0119
**B14** (GeH_2_···OC)	LP (O)→LP* (Ge)	68.01	−0.0197	0.0158	0.0568	0.0003	0.0139	−0.0137
**B15** (SnH_2_···OC)	LP (O)→LP* (Sn)	49.78	−0.0150	0.0130	0.0493	0.0007	0.0116	−0.0109
**B16** (PbH_2_···OC)	LP (O)→LP* (Pb)	49.45	−0.0148	0.0142	0.0574	0.0010	0.0133	−0.0123
**B17** (SiH_2_···C_6_H_6_)	π (C = C)→LP* (Si)	221.92	−0.0726	0.0374	0.0137	−0.0104	0.0139	−0.0243
**B18** (GeH_2_···C_6_H_6_)	π (C = C)→LP* (Ge)	185.55	−0.0719	0.0335	0.0501	−0.0053	0.0178	−0.0231
**B19** (SnH_2_···C_6_H_6_)	π (C = C)→LP* (Sn)	106.21	−0.0611	0.0249	0.0518	−0.0019	0.0149	−0.0168
**B20** (PbH_2_···C_6_H_6_)	π (C = C)→LP* (Pb)	82.85	−0.0586	0.0251	0.0634	−0.0010	0.0169	−0.0179

**Table 6:** Decomposition of the total interaction energy (*E*_tot_) for type-B complexes into electrostatic (*E*_ele_), induction (*E*_ind_), dispersion (*E*_disp_), and exchange (*E*_ex_) energy terms. All energies in kJ/mol. The relative values in percent represent the contribution of electrostatic, induction, and dispersion energy terms to the sum of all the three energy terms.

Complex	*E*_ele_	%*E*_ele_	*E*_ind_	%*E*_ind_	*E*_disp_	%*E*_disp_	*E*_ex_	*E*_tot_
**B1** (SiH_2_···N_2_)	−98.06	46.2	−76.66	36.2	−37.24	17.6	187.68	−24.29
**B2** (GeH_2_···N_2_)	−66.13	47.8	−44.06	31.9	−28.13	20.3	118.96	−19.35
**B3** (SnH_2_···N_2_)	−36.74	45.4	−24.16	29.9	−20.02	24.7	64.33	−16.59
**B4** (PbH_2_···N_2_)	−30.68	46.1	−18.14	27.2	−17.81	26.7	52.04	−14.59
**B5** (SiH_2_···HCN)	−207.75	53.4	−129.79	33.4	−51.54	13.2	323.70	−65.38
**B6** (GeH_2_···HCN)	−158.71	55.8	−85.23	29.9	−40.76	14.3	231.74	−52.96
**B7** (SnH_2_···HCN)	−101.24	53.7	−55.97	29.7	−31.22	16.6	140.82	−47.61
**B8** (PbH_2_···HCN)	−78.58	55.3	−37.87	26.6	−25.71	18.1	101.57	−40.59
**B9** (SiH_2_···CO)	−301.29	49.1	−237.63	38.7	−74.40	12.2	516.90	−96.43
**B10** (GeH_2_···CO)	−256.48	52.7	−169.50	34.9	−60.53	12.4	419.05	−67.47
**B11** (SnH_2_···CO)	−95.51	46.5	−74.03	36.0	−35.91	17.5	162.77	−42.68
**B12** (PbH_2_···CO)	−68.43	48.3	−45.52	32.1	−27.76	19.6	108.72	−32.98
**B13** (SiH_2_···OC)	−15.80	39.2	−11.58	28.7	−12.92	32.1	32.27	−8.03
**B14** (GeH_2_···OC)	−16.97	40.1	−11.91	28.2	−13.42	31.7	34.53	−7.77
**B15** (SnH_2_···OC)	−13.25	39.6	−8.86	26.5	−11.33	33.9	25.67	−7.77
**B16** (PbH_2_···OC)	−12.54	39.5	−8.11	25.6	−11.08	34.9	24.08	−7.65
**B17** (SiH_2_···C_6_H_6_)	−113.99	41.9	−94.84	34.9	−63.03	23.2	231.70	−40.17
**B18** (GeH_2_···C_6_H_6_)	−104.88	43.2	−79.55	32.7	−58.44	24.1	206.24	−36.62
**B19** (SnH_2_···C_6_H_6_)	−65.04	38.5	−54.21	32.0	−49.91	29.5	135.06	−34.11
**B20** (PbH_2_···C_6_H_6_)	−58.31	38.2	−46.86	30.7	−47.36	31.1	121.89	−30.64

## Data Availability

Not applicable.

## Supplementary Materials

The following supporting information can be downloaded at: https://www.mdpi.com/article/10.3390/molecules28062577/s1. Figure S1. Correlation between the |*E*_int_| and *G* or |*V*| values for the SiH_2_···T’H_3_F system, and Figure S2. Correlation between the |*E*_int_| and *G* or |*V*| values for the TH_2_···CO system.
