# Supplementary material for: Tetrel-Bond Interactions Involving Metallylenes TH2 (T = Si, Ge, Sn, Pb): Dual Binding Behavior
Source: Molecules. 2023 Mar 12;28(6):2577. doi: 10.3390/molecules28062577 (PMC10051284; doi:10.3390/molecules28062577)
Supplement: Supplementary file 1 [file molecules-28-02577-s001.zip › molecules-2269342-supplementary.pdf]

Tetrel-bond interactions involving metallylenes TH_2 (T = Si, Ge, Sn, Pb): Dual binding behavior

Yishan Chen*,
Lifeng Yao, Fan Wang

School of Chemistry & Environmental Science, Qujing Normal University, Qujing 655011, Yunnan, China

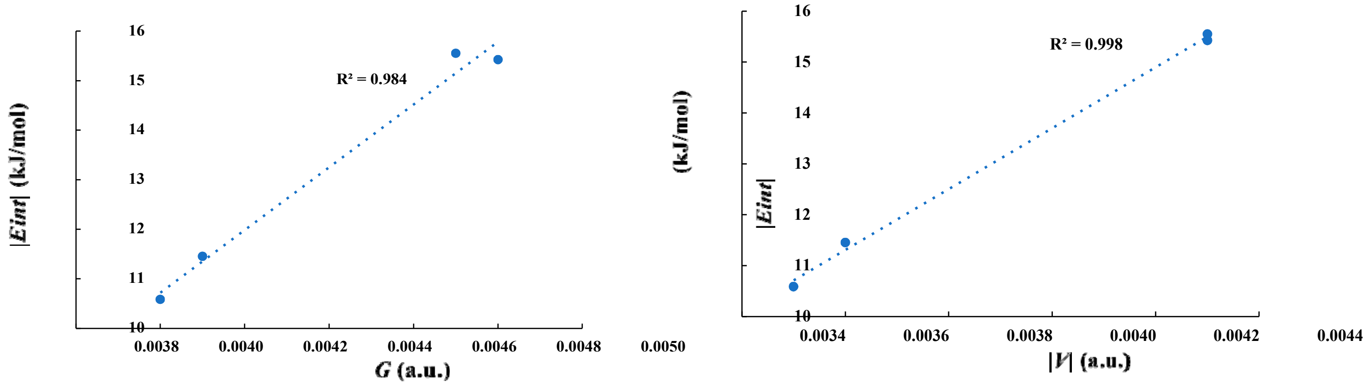

Figure S1. Correlation between the $|E_{\mathrm{int}}|$ and G or $|V|$ values for the $SiH_2\cdots T'H_3F$ system.

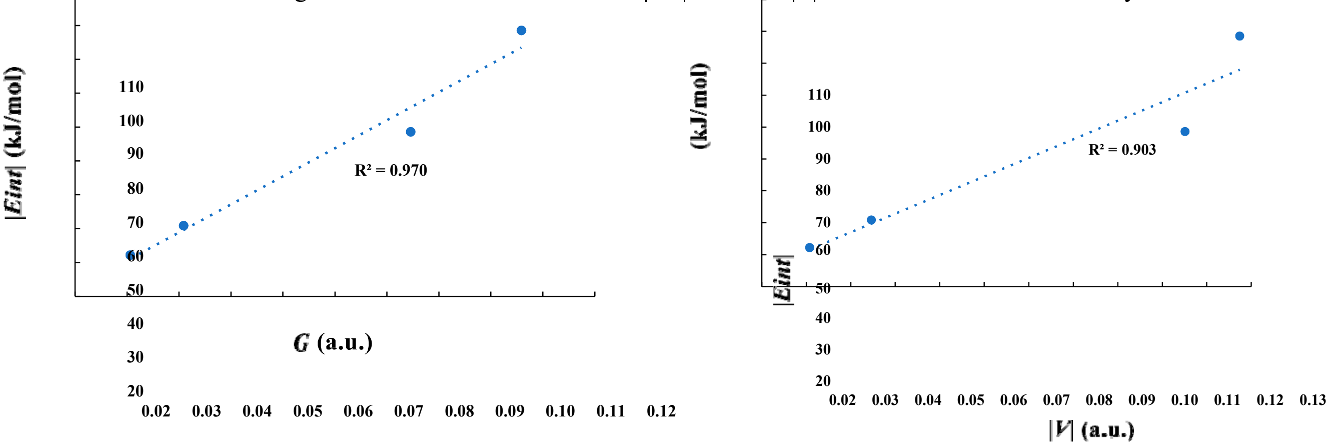

Figure S2. Correlation between the $|E_{\mathrm{int}}|$ and G or $|V|$ values for the $TH_2\cdots CO$ system.
